# Exaggerated IL-15 and Altered Expression of foxp3+ Cell-Derived Cytokines Contribute to Enhanced Colitis in Nlrp3−/− Mice

**DOI:** 10.1155/2016/5637685

**Published:** 2016-08-17

**Authors:** Simon A. Hirota, Aito Ueno, Sarah E. Tulk, Helen M. Becker, L. Patrick Schenck, Mireille S. Potentier, Yan Li, Subrata Ghosh, Daniel A. Muruve, Justin A. MacDonald, Paul L. Beck

**Affiliations:** ^1^Department of Physiology and Pharmacology, University of Calgary, Calgary, AB, Canada T2N 4N1; ^2^Department of Microbiology, Immunology & Infectious Diseases, University of Calgary, Calgary, AB, Canada T2N 4N1; ^3^Department of Medicine, University of Calgary, Calgary, AB, Canada T2N 4N1; ^4^Department of Biochemistry & Molecular Biology, University of Calgary, Calgary, AB, Canada T2N 4N1

## Abstract

The pathogenesis of Crohn's disease (CD) involves defects in the innate immune system, impairing responses to microbes. Studies have revealed that mutations NLRP3 are associated with CD. We reported previously that Nlrp3−/− mice were more susceptible to colitis and exhibited reduced colonic IL-10 expression. In the current study, we sought to determine how the loss of NLRP3 might be altering the function of regulatory T cells, a major source of IL-10. Colitis was induced in wild-type (WT) and Nlrp3−/− mice by treatment with dextran sulphate sodium (DSS). Lamina propria (LP) cells were assessed by flow cytometry and cytokine expression was assessed. DSS-treated Nlrp3−/− mice exhibited increased numbers of colonic foxp3+ T cells that expressed significantly lower levels of IL-10 but increased IL-17. This was associated with increased expression of colonic IL-15 and increased surface expression of IL-15 on LP dendritic cells. Neutralizing IL-15 in Nlrp3−/− mice attenuated the severity of colitis, decreased the number of colonic foxp3+ cells, and reduced the colonic expression of IL-12p40 and IL-17. These data suggest that the NLRP3 inflammasome can regulate intestinal inflammation through noncanonical mechanisms, providing additional insight as to how NLRP3 variants may contribute to the pathogenesis of CD.

## 1. Introduction

The pathogenesis of the inflammatory bowel disease (IBD), Crohn's disease (CD), and ulcerative colitis (UC) is unknown; however the current paradigm suggests that aberrant immune interactions between genetically susceptible individuals and environmental factors trigger the chronic inflammatory response [1]. Although it is apparent that, through dysregulated T cell function, the adaptive immune system drives chronic inflammation in IBD, it has been hypothesized that deficiencies in the innate immune system that render it hyporesponsive to the intestinal microbiota also play a role in the initiation of the inflammatory response [2]. Indeed, genome-wide association studies have reported that loss-of-function mutations in the genes encoding microbial receptors of the innate immune system, such as NOD2 and NLRP3 (nucleotide-binding, leucine-rich repeat (NLR) family, pyrin domain containing 3), are associated with an increased risk for CD [3, 4].

NLRP3, a member of the NLR subfamily of innate immune receptors and component of the inflammasome, is involved in the caspase-1-dependent processing of pro-IL-1*β* and pro-IL-18 in response to a variety of pathogens and endogenous danger signals [5]. In a previous study, we reported that Nlrp3−/− mice were more susceptible in experimental models of colitis [6], exhibiting decreased intestinal barrier function, altered expression of antimicrobial peptides, and a unique intestinal microbiota. Others have reported that the NLRP3 inflammasome is integral in the maintenance of mucosal integrity through its processing of pro-IL-18 [7, 8]. More recently, the NLRP6 inflammasome, whose activating ligands have yet to be identified, has been implicated as a regulator of intestinal mucosal homeostasis by shaping the intestinal microbiota and enhancing mucosal regeneration following colitis-associated injury [9–12]. Taken together these data highlight the importance of inflammasomes in the regulation of intestinal homeostasis.

In addition to the broad changes in innate mucosal immune function, our previous report indicated that Nlrp3−/− mice exhibited reduced colonic IL-10 and TGF-*β*, suggesting that alterations in inflammasome function may trigger profound changes in the function of additional cell types, such as T cells [6]. Interestingly, T cell-inflammasome interactions have been reported in a number of different organ systems. Interferon-*β*-primed memory T cells can inhibit inflammasome signaling in monocytes by downregulating the P2X_7_ receptor [13], whereas T cell-derived IL-22 enhances inflammasome activation in adipose-derived macrophages [14]. On the other hand, inflammasomes have been implicated in the regulation of T cell function, enhancing T helper type-1 (Th1) and T helper type-17 (Th17) responses in experimental autoimmune encephalomyelitis [15]. Furthermore, NLRP3 activation has been associated with the mechanisms that drive T helper type-2 (Th2) responses and allergic sensitization in the airways [16]. Taken together, it is apparent that there is a complex interplay between inflammasome signaling and T cells, suggesting reciprocal modulation of function.

In the gastrointestinal tract, T cell dysregulation is associated with enhanced mucosal inflammation. In animal models, deletion of IL-10, a prototypical T regulatory cell- (Treg-) derived immunomodulatory cytokine, results in spontaneous intestinal inflammation [17]. In human IBD, Th17 cells are thought to contribute to intestinal inflammation as the expression of IL-17 is significantly elevated in the inflamed mucosa of CD patients [18]. Furthermore, other reports suggest a disruption in the delicate equilibrium between Treg populations and Th17 cells in the mucosa of IBD patients [19]. Additionally, polymorphisms in the gene encoding the receptor for IL-23, a cytokine that is critical in the development of the Th17 lineage, are associated with a number of inflammatory disorders, including IBD [20, 21].

Given the conclusions from the aforementioned studies, and the data from our previous report indicating that the deletion of Nlrp3 was associated with reduced colonic IL-10 and more severe colitis, in the current study we sought to test the hypothesis that the NLRP3 inflammasome contributes to the maintenance of intestinal homeostasis through its ability to regulate Treg cell function. Herein we report that colitic Nlrp3−/− mice exhibit increased numbers of foxp3+ Treg cells in the colonic mucosa, but these cells display reduced IL-10 expression compared to cells isolated from wild-type (WT) mice. Furthermore, foxp3+ Treg cells isolated from colitic Nlrp3−/− mice display enhanced plasticity towards Th17 with increased expression of IL-17. Nlrp3−/− dendritic cells (DCs) exhibit enhanced expression of IL-15, a cytokine reported to drive foxp3+ cell proliferation while inhibiting Treg cell function [22, 23]. Lastly, inhibition of IL-15 signaling with a neutralizing antibody protected Nlrp3−/− from colitis, an effect that was associated with a reduction in the expression of IL-17 in colonic tissue.

## 2. Methods

### 2.1. Animals

All experiments were approved by the Health Sciences Animal Care Committee, University of Calgary, and conform to the guidelines set by the Canadian Council on Animal Care. Wild-type (WT) and Nlrp3−/− (both on a C57Bl/6 background) mice were generated from Nlrp3+/− mice (a gift from the late Dr. Tschopp, University of Lausanne, Dorigny, Switzerland) and males used between 8 and 10 weeks of age.

### 2.2. Induction and Assessment of Colitis

Colitis was induced by the addition of dextran sulphate sodium (DSS; 3.5% w/v, molecular weight, 40,000; ICN Biomedical; Solon, OH, USA) to the drinking water as described previously [6]. Animals were assessed and body weights recorded daily. Hematocrit values were assessed as an index of blood loss. Tissue damage was assessed in a blinded fashion using parameters that we have described previously [6].

### 2.3. Assessment of Tissue Myeloperoxidase Activity and Cytokine Levels

Myeloperoxidase (MPO) activity was determined following a published protocol [24]. MPO activity was reported as units/mg tissue, where one unit of MPO was defined as the amount of enzyme needed to degrade 1 *μ*mol of H_2_O_2_ per minute at room temperature. Cytokine levels were assessed with a Luminex XMap bead-based cytokine array as we have described previously [6]. Colonic segments stored at −80°C were placed in ice-cold lysis buffer (250 *μ*L/25 mg tissue; 10 mM Tris pH 7.5, 1% NP-40, 150 mM NaCl, and protease inhibitor cocktail), minced with dissection scissors and then homogenized, decanted into sterile Eppendorf tubes, and centrifuged at 12,000 rpm for 15 minutes. The supernatant was then filtered through an Ultrafree-MC centrifugal filter device (Millipore Corp.) with centrifugation at 12,000 rpm for 5 minutes. The protein concentration of all samples was equalized by dilution with lysis buffer. To assess cytokine levels, prelabeled beads were mixed and added to each well along with samples prepared in triplicate at two different total protein concentrations (10 and 35 mg total protein). Samples were incubated with beads for 2 hours and then washed 3 times and incubated with secondary antibodies for 1 hour. Following incubation, wells were washed and then beads assessed in the Luminex 200 System. Mean fluorescence values were measured and cytokine concentrations calculated using the prepared standards.

### 2.4. Immunofluorescent Staining for foxp3 and IL-15

Paraffin embedded tissue was stained as described previously [25]. Sections were incubated with anti-mouse foxp3 (BioLegend Cat.# 320001; San Diego, CA, USA) or biotin-conjugated anti-mouse IL-15 (R&D Systems Cat.# BAF 447; Burlington, ON, CAN). Cell counts were performed in a blinded fashion under fluorescent microscopy and expressed as the number of positive cells per high-powered field (HPF; 40x).

### 2.5. Neutralizing Anti-IL-15 Antibody Treatment

Nlrp3−/− mice were treated daily with a neutralizing anti-IL-15 antibody or rat IgG isotype control antibody (eBioscience, 5 *μ*g i.p. daily in 100 *μ*L of sterile saline; San Diego, CA, USA) as described by Watkins et al. [26], starting one day before DSS exposure. Colitis severity was assessed as described previously [6].

### 2.6. Flow Cytometric Analysis of Freshly Isolated Lamina Propria Cells and Splenocytes

To assess dendritic cell and T cell subsets, leukocytes were extracted from the lamina propria (LP) of the colon as previously described [25]. Splenocytes were isolated with mechanical disruption by macerating the spleen through a 100 *μ*m cell sieve. Isolated cells were washed with Stabilization Buffer (PBS containing 0.2% (w/v) BSA and 0.1% (w/v) sodium azide) followed by centrifugation (1000 rpm, 10 minutes). After blocking with CD32/CD16 antibodies (clone 2.4GK, BD Biosciences; San Diego, CA, USA), cells were labeled with anti-mouse CD3 (clone 17A2) and CD4 (clone GK1.5) and foxp3 (clone FJK-16s) antibodies (eBioscience; San Diego, CA, USA). Intracellular foxp3 expression was detected using the Foxp3 Stain Kit (eBioscience; San Diego, CA, USA) as per the manufacturer's protocol. DCs were stained with anti-mouse CD11c (clone HL3), CD45R/B220 (clone RA3-6B2), I-A/I-E (class II MHC, clone 2G9), CD8a (clone 53-6.7), and CD11b (clone M1/70) antibodies (all from BD Biosciences Pharmingen; Mississauga, ON, CAN). Our gating strategy for DC experiments is as follows: first, a side scatter low DC/macrophage gate was employed. Next, DCs were defined as the CD11c and I-A/I-E expressing population. DC subsets were characterized by further gating to define plasmacytoid DC as B220 high and CD11b low; myeloid DC as B220 negative and CD11b high; and lymphoid DC as CD8a expressing populations.

### 2.7. Flow Cytometric Assessment of Intracellular Cytokines

To detect intracellular cytokine expression, isolated splenocytes as well as LP cells were cultured with phorbol 12-myristate 13-acetate (PMA, 5 ng/mL, Sigma-Aldrich; Oakville, ON, CAN) and ionomycin (15 nM, Sigma-Aldrich; Sigma-Aldrich; Oakville, ON, CAN) for 1 hour followed by incubation with Golgistop (Monensin, BD Biosciences; San Diego, CA, USA) for 12 hours (5% CO_2_ and 95% humidity at 37°C). Surface staining was performed with CD4 and CD25 (clone PC61, BD Biosciences; San Diego, CA, USA) antibody, followed by a wash in Stabilization Buffer. Intracellular cytokine staining was performed using the Cytofix/Cytoperm Kit (BD Biosciences; San Diego, CA, USA) as per the manufacturer's protocol. The following primary antibodies were used: fluoroconjugated rat IgG1 anti-mouse IL-10 (clone JES5-16E3; BD Biosciences; San Diego, CA, USA) and IL-17A (clone TC11-18H10; BD Biosciences; San Diego, CA, USA).

### 2.8. Generation of Bone Marrow-Derived DCs

Briefly, femurs and tibias of the mice were dissected, and the marrow flushed with complete RPMI media. The bone marrow (BM) cells were pelleted by centrifugation (1300 rpm, 6 minutes) at 4°C and then plated on to noncoated Petri dishes for 10 days at a concentration of 10^8^ cells/dish with recombinant mouse Flt3 ligand (Flt3L, 100 ng/mL, eBioscience). The Flt3L-containing media was changed every 3 days.

### 2.9. Statistics

All values are represented as the mean ± standard error of the mean (SEM). Simple, two-group comparisons were analyzed using Student's *t*-test. For multiple comparisons, a two-way ANOVA was utilized followed by a Newman-Keuls post hoc test.

## 3. Results

### 3.1. Nlrp3−/− Mice Exhibit Increased Susceptibility to Experimental Colitis, an Effect Associated with Reduced IL-10 Expression but Increased Numbers of Colonic foxp3+/CD4+ Cells

**Figure 1:**
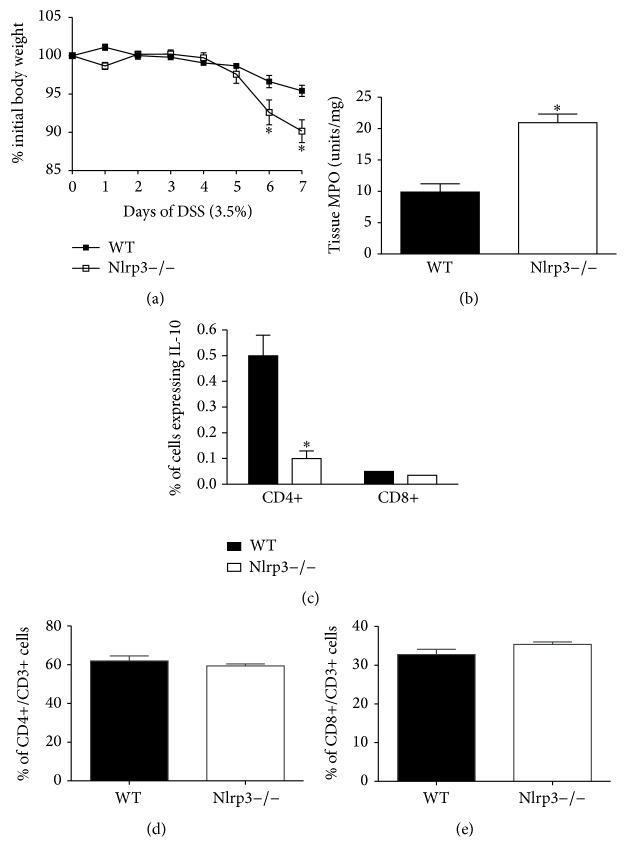
Nlrp3−/− mice exhibit enhanced susceptibility to DSS-induced colitis, a phenotype that associated with reduced IL-10 expression. (a) Changes in body weight during a 7-day course of DSS (3.5% w/v in drinking water) in WT and Nlrp3−/− mice. *∗* denotes *p* < 0.05 compared to WT; *n* = 6/group. (b) Colonic tissue myeloperoxidase (MPO) levels assessed in WT and Nlrp3−/− mice on day 7 of DSS. *∗* denotes *p* < 0.05 compared to WT mice; *n* = 6/group. (c) Flow cytometric analysis of IL-10 expression in CD4+ and CD8+ T cells from the LP of WT and Nlrp3−/− mice on day 7 of DSS. *∗* denotes *p* < 0.05 compared to WT mice; *n* = 6/group. (d) Flow cytometric analysis of total CD4+/CD3+ and (e) CD8+/CD4+ cells isolated from the LP of WT and Nlrp3−/− mice on day 7 of DSS; *n* = 6/group.

Over the 7-day course of DSS treatment, Nlrp3−/− mice exhibited significantly greater weight loss (Figure 1(a)) and increased colonic MPO levels (assessed on day 7 of DSS; Figure 1(b)), indicating enhanced granulocytic inflammatory infiltration. In our previous characterization of the Nlrp3−/− mice, we reported that these mice exhibited reduced colonic IL-10 expression during the inflammatory phase of DSS-induced colitis [6]. To determine whether the loss of NLRP3 inflammasome function and the resulting increased susceptibility to experimental colitis were driven by changes in T cell phenotype, we assessed the expression of IL-10 in CD4+ and CD8+ T cell populations isolated from the colonic LP. Interestingly, we observed reduced IL-10 expression in CD4+, but not CD8+ T cells isolated from Nlrp3−/− mice on day 7 of DSS when compared to those isolated from WT mice (Figure 1(c)). There were no differences in total CD4+/CD3+ or CD8+/CD3+ cells between colitic WT and Nlrp3−/− mice (Figures 1(d) and 1(e)).

**Figure 2:**
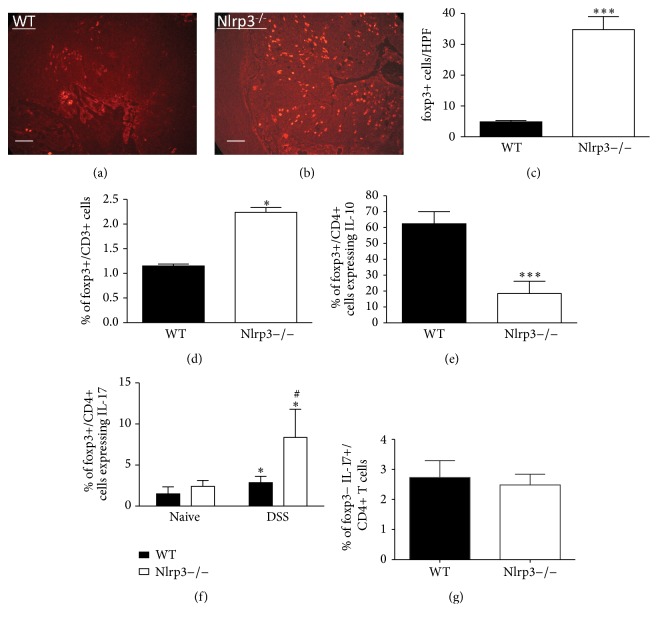
DSS-treated Nlrp3−/− mice exhibit increased numbers of colonic foxp3+ cells that exhibit reduced IL-10 but increased IL-17 expression. Representative images of (a) WT and (b) Nlrp3−/− colonic sections stained for foxp3; scale bar = 50 microns. (c) Quantification of foxp3+ cells in WT versus Nlrp3−/− mice on following 7 days of DSS exposure. Data are expressed as the number of foxp3+ cells per high-powered field (HPF). *∗∗∗* denotes *p* < 0.005 compared to WT mice; *n* = 6/group; 4 HPF/mouse. (d) Flow cytometric analysis of foxp3+/CD3+ cells isolated from the LP of WT versus Nlrp3−/− mice on following 7 days of DSS exposure. *∗* denotes *p* < 0.05 compared to WT mice; *n* = 6/group. (e) Flow cytometric analysis of IL-10 expression in foxp3+/CD4+ T regulatory cells isolated from the LP of WT and Nlrp3−/− mice on day 7 of DSS. *∗∗∗* denotes *p* < 0.005 compared to WT nice; *n* = 5/group. (f) IL-17 expression in foxp3+/CD4+ cells isolated from the LP of naive and DSS-treated WT and Nlrp3−/− mice. *∗* denotes *p* < 0.05 compared to naive mice; # denotes *p* < 0.05 compared to WT mice; *n* = 6/group. (g) IL-17 expression in foxp3−/CD4+ cells isolated from the LP DSS-treated WT and Nlrp3−/− mice; *n* = 6/group.

Given the dramatic reduction in IL-10 expression in the CD4+ cells isolated from Nlrp3−/− mice, we sought to assess the number of foxp3+/CD4+ cells in the colonic tissues from DSS-exposed WT and Nlrp3−/− mice. Interestingly, despite decreased IL-10 expression in colitic Nlrp3−/− mice, we observed a significant increase in the number of foxp3+ cells in the colonic mucosa of Nlrp3−/− mice following a 7-day course of DSS (Figures 2(a) and 2(b), quantified in 2(c)). This was confirmed with flow cytometric analysis of LP cells (foxp3+/CD4+ cells) isolated from WT and Nlrp3−/− on day 7 of DSS (Figure 2(d)).

### 3.2. DSS-Treated Nlrp3−/− Mice Display Reductions in foxp3+/IL-10 Cells but an Increase in Number of foxp3+/IL-17 Cells

Since we observed a paradoxical increase in the number of foxp3+ cells in Nlrp3−/− mice, despite reduced colonic/foxp3+ cell IL-10 expression, we next sought to determine whether the foxp3+/CD4+ cells isolated from colitic Nlrp3−/− exhibit alterations in cytokine expression. Flow cytometric analysis of foxp3+/CD4+ cells isolated from DSS-treated Nlrp3−/− mice revealed reduced IL-10 expression in the Nlrp3−/− cells, compared to WT (Figure 2(e)). Further assessment of the foxp3+/CD4+ population of cells revealed a significant increase in the expression of IL-17 in cells isolated from DSS-exposed Nlrp3−/− mice, compared to those isolated from WT mice (Figure 2(f)), an effect that was not evident in naive, non-DSS-treated mice. No differences in IL-17 expression were observed in foxp3−/CD4+ cells isolated from DSS-treated Nlrp3−/− and WT mice (Figure 2(g)).

### 3.3. Nlrp3−/− Mice Exhibit Enhanced IL-15 Expression

**Figure 3:**
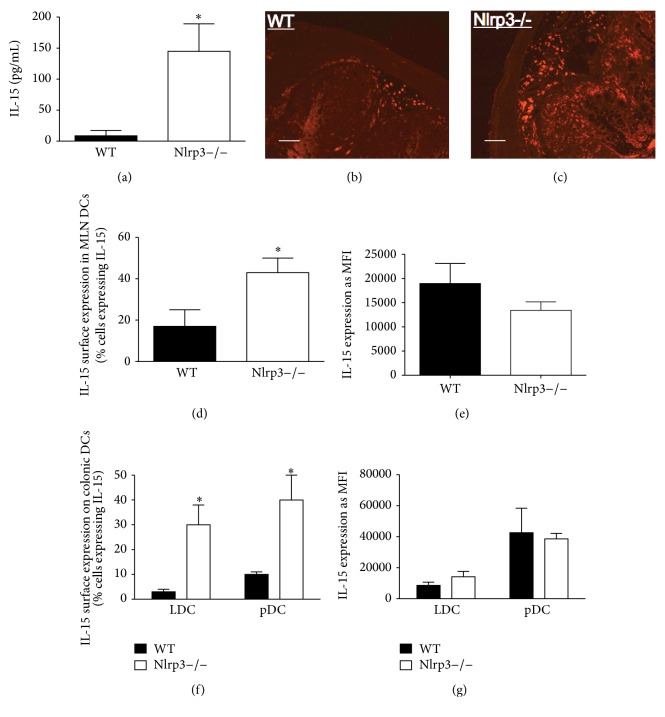
Nlrp3−/− mice exhibit increased numbers of IL-15 expressing DCs in the MLN and colonic following a 7-day course of DSS. (a) The quantification of IL-15 in colonic tissue isolated from WT and Nlrp3−/− on day 7 of DSS. *∗* denote *p* < 0.05 compared to WT; *n* = 5/group. Representative images of (b) WT and (c) Nlrp3−/− colonic sections stained for IL-15; scale bar = 100 microns. Flow cytometric analysis of IL-15 expression on the surface of all DCs isolated from the MLNs of WT and Nlrp3−/− on day 7 of DSS, expressed as a percentage of IL-15 expressing cells (d) and mean fluorescence intensity (MFI) per cell (e); *∗* denotes *p* < 0.05 compared to WT; *n* = 6/group. Flow cytometric analysis of IL-15 expression on the surface of lymphoid (CD11c+/CD8+; LDC) and plasmacytoid (CD11b+/B220+/CD11c−; pDC) DCs isolated from the LP of WT and Nlrp3−/− mice on day 7 of DSS, expressed as a percentage of IL-15 expressing cells (f) and MFI per cell (g); *∗* denotes *p* < 0.05 compared to WT; *n* = 5/group.

Recent reports have highlighted the existence of foxp3+/IL-17+ cells in the tissue [27] and peripheral blood [28] of patients with IBD. Furthermore, Ferretti et al. reported that foxp3+ cells could be driven to increase the expression of IL-17 through the action of IL-15 [29]. Thus, we sought to determine if the aberrant cytokine expression observed in foxp3+ cells isolated from colonic tissue of DSS-treated Nlrp3−/− mice could be driven by increased expression of IL-15. Indeed, DSS-treated Nlrp3−/− displayed enhanced colonic IL-15 expression when assessed by a Luminex bead-based assay (Figure 3(a)), an observation that was confirmed by immunostaining colonic sections for IL-15 (Figures 3(b) and 3(c)). Previous reports have suggested that DCs are a significant source of IL-15 in intestinal tissue [30]. Expressed on the surface of DCs, IL-15 can modulate T cell function and can trigger the proliferation of foxp3+ T cells [30–32]. Thus, we sought to assess the expression of IL-15 on DCs isolated from the colonic LP of DSS-treated mice. Interestingly, we observed an increase in the number of IL-15 expressing DCs isolated from the mesenteric lymph nodes (MLNs) of Nlrp3−/− mice on day 7 of DSS (Figure 3(d)), without any difference in mean fluorescence intensity (MFI; Figure 3(d)). We also observed an increase in the number of IL-15 expressing lymphoid (LDC) and plasmacytoid (pDC) DCs isolated from Nlrp3−/− mice on day 7 of DSS (Figure 3(f)) compared to DCs isolated from WT mice, without any difference in MFI between strains (Figure 3(g)).

### 3.4. BM-Derived DCs from Nlrp3−/− Mice Exhibit Enhanced Frequency of IL-15 Expression

**Figure 4:**
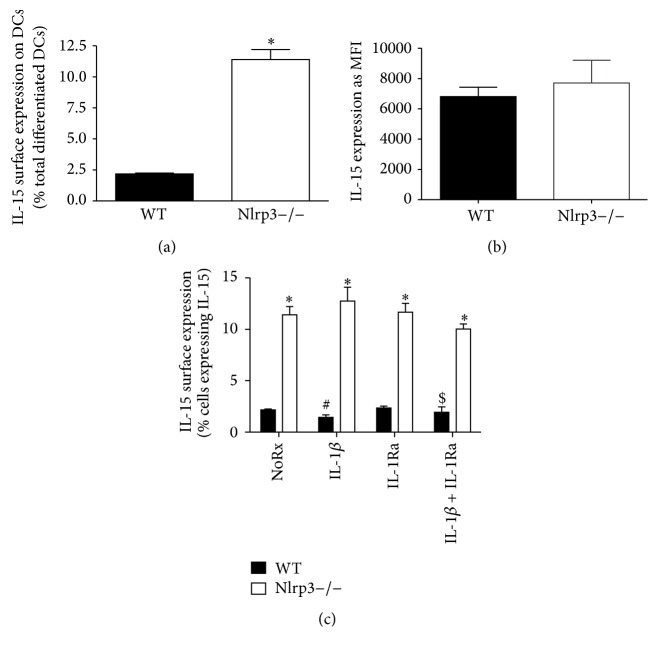
BM-derived DCs from Nlrp3−/− mice exhibit increased frequency of IL-15 expression an effect that cannot be normalized by exogenous IL-1*β*. (a) Flow cytometric analysis of IL-15 expression on the surface of WT and Nlrp3−/− DCs differentiated from BM progenitor cells for 10 days with Flt3L (100 ng/mL), expressed as a percentage of IL-15 expressing cells (a) and MFI per cell (b). (c) Flow cytometric analysis of IL-15 surface expression on WT and Nlrp3−/− DCs differentiated from BM progenitor cells for 10 days with Flt3L (100 ng/mL) and treated with IL-1*β* (100 ng/mL) and/or recombinant IL-1 receptor antagonist (IL-1Ra; 100 *μ*g/mL) for 12 hours. *∗* denotes *p* < 0.05 compared to WT mice; # denotes *p* < 0.05 compared to WT, NoRx; $ denotes *p* < 0.05 compared to WT-IL-1*β*; *n* = 5/group.

Following our observation that DCs isolated from the colonic LP and MLNs of DSS-treated Nlrp3−/− mice displayed increased IL-15 surface expression, we sought to determine if this was a property intrinsic to the cells themselves, independent of their environment. Thus, we differentiated BM-derived progenitor cells isolated from WT and Nlrp3−/− mice into DCs by culturing them for 10 days in the presence of Flt3 ligand (Flt3L; 100 ng/mL). Indeed, a greater number of DCs generated from BM isolated from Nlrp3−/− mice displayed IL-15 surface expression when compared to cells differentiated from WT BM (Figure 4(a)), an effect that was not associated with an increase in IL-15-MFI (Figure 4(b)). To determine if the differences in IL-15 expression were due to the loss of inflammasome-dependent IL-1*β* production, we treated WT and Nlrp3−/− DCs with exogenous IL-1*β* (100 ng/mL) in the presence and absence of recombinant IL-1 receptor antagonist (IL-1Ra; 100 *μ*g/mL). The number of WT DCs treated with IL-1*β* displaying IL-15 expression was significantly reduced (compared to the vehicle control, NoRx), an effect that was blocked with coincubation with IL-1Ra. In contrast, Nlrp3−/− DCs exhibited no significant changes in response to any of the treatment conditions (Figure 4(c)).

### 3.5. Neutralizing IL-15 Protects Nlrp3−/− from Intestinal Inflammation and Damage in the DSS Model of Colitis, an Effect That Is Associated with Reduced Colonic IL-17

**Figure 5:**
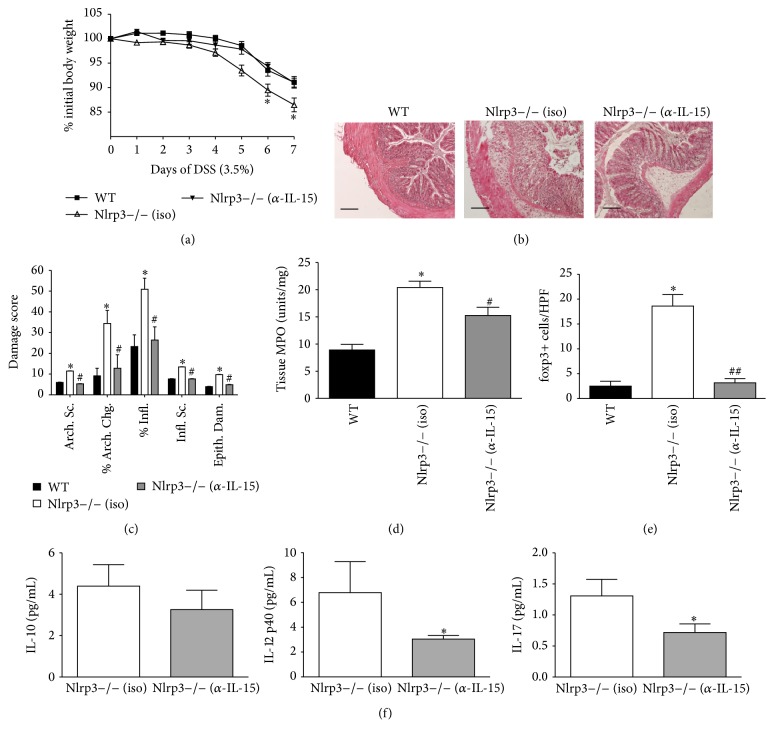
A neutralizing anti-IL-15 antibody normalizes the sensitivity of Nlrp3−/− mice to DSS and reduces colonic IL-17 expression. (a) Changes in body mass in WT mice, Nlrp3−/− mice treated with 5 *μ*g IgG isotype (iso) control, or Nlrp3−/− mice treated with an anti-IL-15 (*α*-IL-15) antibody through a 7-day course of DSS (3.5% w/v). *∗* denotes *p* < 0.05 compared to WT and Nlrp3−/−  *α*-IL-15; *n* = 8/group. (b) Representative hematoxylin and eosin stained colonic sections (scale bar = 100 microns) and (c) blind assessment of tissue damage and inflammation in WT mice, Nlrp3−/− mice treated with 5 *μ*g IgG isotype (iso) control, or Nlrp3−/− mice treated with an anti-IL-15 (*α*-IL-15) antibody on day 7 of DSS. *∗* denotes *p* < 0.05 compared to WT mice; # denotes *p* < 0.05 compared to Nlrp3−/− mice treated with the IgG isotype control antibody; *n* = 8/group. (d) Assessment of colonic tissue MPO levels and (e) colonic foxp3+ cells/HPF in WT mice, Nlrp3−/− mice treated with an IgG isotype (iso) control antibody, and Nlrp3−/− mice treated with a neutralizing anti-IL-15 (*α*-IL-15) antibody on day 7 of DSS. *∗* denotes *p* < 0.05 compared to WT mice; # denotes *p* < 0.05 compared to Nlrp3−/− mice treated with the IgG isotype control antibody; ## denotes *p* < 0.005 compared to Nlrp3−/− mice treated with the IgG isotype control antibody; *n* = 8/group. (f) The expression of IL-10, IL-12p40, and IL-17 in colonic tissue isolated from Nlrp3−/− mice treated with an IgG isotype (iso) control antibody and Nlrp3−/− mice treated with a neutralizing anti-IL-15 (*α*-IL-15) antibody on day 7 of DSS. *∗* denotes *p* < 0.05 compared to Nlrp3−/− mice treated with an IgG isotype (iso) control antibody; *n* = 8/group.

To further assess the role of IL-15 in the increased susceptibility of Nlrp3−/− to DSS-induced colitis, we employed an antibody-based neutralization strategy. Nlrp3−/− mice were treated with either a neutralizing anti-IL-15 antibody or an IgG isotype control antibody at an equimolar dose (5 *μ*g of antibody/mouse/day). The neutralizing anti-IL-15 antibody significantly reduced the severity of intestinal inflammation induced by DSS in Nlrp3−/− mice as indicated by attenuated body weight loss, reduced colonic inflammation, and tissue damage (Figures 5(a)–5(c)) and a reduction in colonic tissue MPO (Figure 5(d)), compared to isotype control-treated Nlrp3−/− mice. Furthermore, neutralization of IL-15 significantly reduced the number of foxp3+ cells detected in colonic sections from Nlrp3−/− mice treated with DSS (Figure 5(e)). Lastly, while having no effect on colonic IL-10 (Figure 5(f)), neutralization of IL-15 significantly reduced the expression of IL-12p40 and IL-17 in colonic tissue isolated from Nlrp3−/− on day 7 of DSS compared to isotype control-treated Nlrp3−/− mice.

## 4. Discussion

In the current study, we have continued our assessment of the role of the NLRP3 inflammasome in the regulation of intestinal homeostasis. Herein, we describe how the loss of NLRP3 signaling results in the increased expression of IL-15, an inflammatory cytokine not previously associated with the inflammasome, and changes in T cell phenotypes.

Alterations in the innate immune system have been implicated in the pathogenesis of IBD, especially CD [2]. Studies have reported that mutations in* NLRP3* are associated with an increased risk for developing CD [3, 4]. We and others have reported previously a protective role of the NLRP3 inflammasome in the maintenance of intestinal homeostasis in the context of experimental colitis [6–8, 33]. However, whether the loss of NLRP3 inflammasome function within the intestinal epithelium or mucosal immune cells contributes to the increased susceptibility to colitis is still a subject of debate. Dupaul-Chicoine et al. concluded that the loss of NLRP3 inflammasome signaling, through the deletion of caspase-1 or the adaptor protein ASC, resulted in enhanced sensitivity to DSS-induced colitis [33]. Furthermore, caspase-12−/− mice, which exhibit increased NLRP3 inflammasome signaling, were protected from DSS-induced colitis [33]. The increased sensitivity of caspase-1−/− mice to DSS was nearly completely reversed upon administration of recombinant IL-18, but only partially reversed upon adoptive transfer of WT myeloid cells, suggesting that a loss of inflammasome signaling in nonmyeloid cells was responsible for the enhanced sensitivity to DSS-induced colitis [33]. Similarly, Zaki et al. reported that deletion of NLRP3, caspase-1 or ASC, increased sensitivity to DSS-induced colitis [34], an effect that could be completely reversed by administration of recombinant IL-18. As reported by Dupaul-Chicoine et al., the loss of inflammasome signaling within the nonhematopoietic compartment conferred the susceptibility [34]. Interestingly, Zaki et al. were able to demonstrate that IL-18 production from colonic intestinal epithelial cells was increased following the induction of colitis in WT, but not ASC–/– or caspase-1–/– mice [34]. In contrast to the aforementioned studies, chimeric experiments performed by Allen et al. revealed that the loss of NLRP3 in BM-derived cells enhanced susceptibility to DSS in Nlrp3−/− mice [8]. They reported that both WT and Nlrp3−/− mice receiving Nlrp3−/− bone marrow exhibited enhanced weight loss, increased severity of disease, and elevated histological assessment scores, compared to WT recipient mice [8].

In our previous report, we found that NLRP3 deficiency altered the expression of intestinal epithelial cell-derived antimicrobial peptides, an effect that reduced bacterial killing and was associated with a distinct fecal microbiota profile [6], suggesting that inflammasome signaling within the nonhematopoietic cell compartment may be contributing to the increased susceptibility to DSS-induced colitis. However, in the current study, we found that that NLRP3-deficiency was associated with elevated expression of IL-15 on the surface of DCs, an effect that was associated with increased expression of IL-17 in foxp3+ T cells. Interestingly, our immunofluorescence assessment of DSS-exposed Nlrp3−/− mice revealed IL-15 expression in mucosal immune cells, but little expression in the intestinal epithelium. Furthermore, neutralizing IL-15 normalized the susceptibility of Nlrp3−/− mice to DSS-induced colitis, an effect that was associated with reduced expression of IL-17 in colonic tissues. Taken together, our data suggest that the loss of inflammasome signaling in myeloid cells may contribute to the increased sensitivity to DSS-induced colitis seen in Nlrp3−/− mice.

In the gastrointestinal tract, IL-15 is primarily expressed by DCs and epithelial cells and can regulate the proliferation and function of a variety of immune cells through stimulation of neighboring cells via* trans*-presentation [35, 36]. Studies assessing the mechanisms governing the production of IL-15 have revealed that its expression as a functional protein is regulated at the translational level; however, very little is known about the signals and the mechanisms that drive its expression on the cell surface [35, 36]. In our study, DSS-treated Nlrp3−/− mice exhibited significant increases in colonic tissue levels of IL-15. Immunostaining revealed increased IL-15 expression in the cells of the LP and submucosal compartment, with limited positive staining on the epithelial layer. Further assessment of LP cells isolated from Nlrp3−/− mice revealed that DCs exhibited enhanced surface expression of IL-15. Currently there is no documented link between the NLRP3 inflammasome and the regulation of IL-15 expression. However, acetaminophen-induced liver injury is mediated by the activation of the NLRP3 inflammasome and subsequent production of IL-1*β* [37, 38], whereas the expression of IL-15 is protective in this system [39], suggesting a reciprocal relationship between NLRP3 and IL-15 in this response. In our study, we observed a similar reciprocal relationship between NLRP3 and IL-15, where the expression of NLRP3 protects mice from experimental colitis and its deletion leads to increased IL-15 expression and enhanced intestinal inflammation. In our* in vitro* experiments, exogenous IL-1*β* could reduce the expression of IL-15 in WT DCs but had no effect on Nlrp3−/− DCs. These data are perplexing and suggest that NLRP3 may play a role in the response to cytokines and the downstream regulation of IL-15 expression. Recently it was reported that NLRP3 can regulate intracellular signaling in an inflammasome-independent fashion, suggesting NLR proteins may elicit biological responses through mechanisms beyond their downstream activation of caspase-1 [40]. In this report, TGF-*β*-induced SMAD2/3 phosphorylation was attenuated in NLRP3-deficient cells, an effect that was independent of IL-1*β*, IL-18, caspase-1, or the inflammasome adaptor ASC [40]. Since very little is known about the regulation of IL-15 expression and trafficking to the membrane, future studies will be required to determine how NLRP3 might interact with these processes, independent of its role in inflammasome signaling.

Functionally, IL-15 has been implicated in the pathogenesis of chronic inflammatory disorders including psoriasis, rheumatoid arthritis, and celiac disease [36]. IL-15 has also been implicated in the pathogenesis of IBD, as mucosal mononuclear cells from IBD patients express increased levels of IL-15, and serum IL-15 levels are increased in patients with IBD [41]. Expression of IL-15 transcripts has also been found to be elevated in the inflamed rectal mucosa of IBD patients [41]. Interestingly, Bouchaud et al. reported that the response to anti-TNF (infliximab) in CD patients was associated with a significant decrease in serum levels of IL-15 [42].

In addition to regulating cells of the innate immune system, IL-15 has been reported to inhibit the regulatory capacity of Tregs and induce the polarization of T cells to a Th17 phenotype [22, 23]. IL-15 can drive CD25^High^ foxp3+ cells to become IL-17-secreting cells, an effect that is not modulated by IL-6 or TGF-*β* [23]. In the context of rheumatoid arthritis, IL-15 drives the development and proliferation of Th17 cells in a mechanism that may involve IL-12 as an intermediary [22]. In our* in vivo* studies, neutralization of IL-15 reduced the expression of IL-12p40, the subunit shared by IL-12 and IL-23 [43], in colonic tissues, a response that was associated with normalization of foxp3+ cell numbers and reduced IL-17 expression, suggesting that either IL-12 or IL-23 signaling mediates these responses. In support of this, Depaolo et al. reported that aberrant IL-15 expression in conjunction with retinoic acid can “break” tolerance to dietary luminal antigens through IL-12/23-dependent inhibition of Treg function and the initiation of Th1- and Th17-like responses [30]. Taken together these data suggest that IL-15 may be contributing to the loss in the regulatory, or tolerance, capacity within the mucosal immune system of colitic Nlrp3−/− mice through an IL-12/23-dependent mechanism.

In our* in vivo* studies, we observed that the loss of NLRP3 increased a population of foxp3+ T cells that exhibited enhanced expression of IL-17. In humans, Hovhannisyan et al. reported the existence of a subset of CD4+ cells in the inflamed mucosa of CD patients that shared the phenotype of Th17 and Treg cells. These cells that express both foxp3 and IL-17 were hypothesized to arise during the differentiation of Th17 and Treg cells [27]. We have also previously demonstrated that the prevalence of circulating IL-17 and foxp3 double expressing cells is associated with impaired Treg function, suggesting the increased Treg plasticity towards Th17 in IBD patients [28]. In our current study, increased IL-15 expression in Nlrp3−/− mice was associated with increased numbers of mucosal foxp3+ cells following 7 days of DSS. The foxp3+ T cells isolated from colitic Nlrp3−/− mice expressed significantly less intracellular IL-10 but exhibited enhanced IL-17 expression. Interestingly, the proliferation of foxp3+ cells and the increased colonic tissue expression of IL-17 were reversed when IL-15 was neutralized during DSS exposure.

The role of IL-15 in animal models of intestinal inflammation is controversial. Yoshihara et al. reported that IL-15-deficient mice exhibited resistance to DSS-induced intestinal injury [44]. Furthermore, inhibition of IL-15 signaling, via antibody-mediated blockade of the IL-15 receptor, protected IL-15-overexpressing mice from intestinal damage [45]. In contrast, Obermeier et al. reported neutralization of IL-15 with soluble IL-15 receptor-*α* (IL-15R*α*) aggravated intestinal epithelial tissue damage and increased the expression of colonic IFN*γ* and TNF-*α* in the DSS model of colitis [46]. However, the use of soluble IL-15R*α* to inhibit IL-15 activity remains controversial. Reports suggest that the soluble IL-15R*α* can enhance rather than inhibit IL-15 binding and activity through the IL-2/IL-15R*βγ* subunits [47]. Furthermore, others have reported that the soluble IL-15R*α* can prolong the signaling life of IL-15 and enhance its ability to signal through a pseudo-*trans*-presentation mechanism [47]. In our study, attenuation of IL-15 signaling with anti-IL-15 antibody treatment was protective, suggesting the method used to inhibit blockade of IL-15 signaling may be important when studying its role* in vivo*.

In summary, we have shown that the increased sensitivity of Nlrp3−/− mice to DSS-induced colitis can be normalized by neutralizing IL-15, an effect that decreases the number of mucosal foxp3+ T cells and significantly reduces the colonic expression of IL-12p40 and IL-17. These data suggest that the NLRP3 inflammasome can regulate intestinal inflammation through noncanonical mechanisms, providing additional insight as to how hypofunctional mutations in* NLRP3* may contribute to the pathogenesis of IBD.

## Abbreviations

IBD: Inflammatory bowel disease
CD: Crohn's disease
NLRP3: Nucleotide-binding domain, leucine-rich repeat containing protein family, pyrin domain containing 3
ASC: Apoptotic Speck protein containing a CARD
APC: Antigen presenting cell
DC: Dendritic cell
Th1: T helper type 1
Th17: T helper type 17
DSS: Dextran sulphate sodium
Tregs: T regulatory cell
WT: Wild-type
MPO: Myeloperoxidase
PBS: Phosphate-buffered saline
LP: Lamina propria
BSA: Bovine serum albumin
PMA: Phorbol 12-myristate 13-acetate
Flt3L: Flt 3 ligand
LDC: Lymphoid DC
pDC: Plasmacytoid DC
i.p.: Intraperitoneal.
